# Healthcare pathways and resource use: mapping consequences of ambulance assessment for direct care with alternative healthcare providers

**DOI:** 10.1186/s12873-020-00380-5

**Published:** 2020-10-30

**Authors:** Sofi Varg, Veronica Vicente, Maaret Castren, Peter Lindgren, Clas Rehnberg

**Affiliations:** 1 Department of Learning, Informatics, Management and Ethics, Medical Management Centre, Karolinska Institutet, Stockholm, Sweden; 2 Centre for Health Economics, Informatics and Health Services Research, Stockholm Health Care Services, Stockholm, Sweden; 3 Ambulance Medical Service in Stockholm [Ambulanssjukvården i Storstockholm AB], Stockholm, Sweden; 4 Academic Emergency Medical Services, Stockholm, Sweden; 5 Department of Clinical Science and Education, Karolinska Institutet, Stockholm, Sweden; 6 Emergency Medicine, Helsinki University and Department of Emergency Medicine and Services, Helsinki University Hospital, Helsinki, Finland; 7 The Swedish Institute for Health Economics, Lund, Sweden

**Keywords:** Emergency medical services, Health care costs, Aged, Decision support systems, clinical, Critical pathways, Delivery of health care

## Abstract

**Background:**

A decision system in the ambulance allowing alternative pathways to alternate healthcare providers has been developed for older patients in Stockholm, Sweden. However, subsequent healthcare resource use resulting from these pathways has not yet been addressed. The aim of this study was therefore to describe patient pathways, healthcare utilisation and costs following ambulance transportation to alternative healthcare providers.

**Methods:**

The design of this study was descriptive and observational. Data from a previous RCT, where a decision system in the ambulance enabled alternative healthcare pathways to alternate healthcare providers were linked to register data. The receiving providers were: primary acute care centre or secondary geriatric ward, both located at the same community hospital, or the conventional pathway to the emergency department at an acute hospital. Resource use over 10 days, subsequent to assessment with the decision system, was mapped in terms of healthcare pathways, utilisation and costs for the 98 included cases.

**Results:**

Almost 90% were transported to the acute care centre or geriatric ward. The vast majority arriving to the geriatric ward stayed there until the end of follow-up or until discharged, whereas patients conveyed to the acute care centre to a large extent were admitted to hospital. The median patient had 6 hospital days, 2 outpatient visits and costed roughly 4000 euros over the 10-day period. Arrival destination geriatric ward indicated the longest hospital stay and the emergency department the shortest. However, the cost for the 10-day period was lower for cases arriving to the geriatric ward than for those arriving to the emergency department.

**Conclusions:**

The findings support the appropriateness of admittance directly to secondary geriatric care for older adults. However, patients conveyed to the acute care centre ought to be studied in more detail with regards to appropriate level of care.

## Background

An aging population results in increased healthcare utilisation and expenditure growth [1, 2]. The older population contributes to a high pressure at emergency departments (ED) and many seek emergency care with vague symptoms and multiple conditions, which result in demanding medical assessment and long stays [3–7].

Older patients are more likely to arrive to the ED by ambulance than their younger peers [5, 7, 8], and to prevent inappropriate ED attendance and inpatient admission, interventions could be directed towards these older patients [8]. There are examples of various initiatives to bypass EDs for direct care with alternate providers [9–14]. One approach is to use systems designed to support clinical decision-making, with so called decision support tools, in the ambulance, as these potentially shorten the time to definitive care [15].

In Sweden, the emergency medical services (EMS) have evolved from being a transport organisation to an advanced healthcare provider. As opposed to previously, when standard pathway for all patients was to the ED, EMS personnel now have the potential to convey patients directly to definitive care, and hence bypass EDs [16].

### The intervention

The basis for the present study is a randomised controlled trial (RCT) in Stockholm, Sweden [11]. The aim of that study was to evaluate the feasibility and appropriateness of a prehospital system allowing ambulance nurses to transport older adults directly to geriatric care at a community-based hospital or to an ED.

In the RCT, an intervention was designed where a decision system was used in the ambulance to assess appropriate level of healthcare [11]. Patients aged 65 or older, living in a specific catchment area, without life-threatening symptoms, and requiring ambulance services during daytime, were included and randomised by the dispatch centre, i.e. the respondent of the emergency call. Inclusion criteria were thereafter refined by the ambulance nurse at site, to only assess patients with any of 11 different predefined medical conditions to an appropriate level of care. The conditions were: urinary disorders (with or without catheters), chronic obstructive pulmonary disease, pneumonia, dizziness, diabetes mellitus (excluding hypoglycaemia), frailty, feverishness, fall, hypotension, hip trauma (without clinical suspicion of femur fracture), and back pain or contusion [11, 17]. The conditions were chosen as they were all common conditions that fulfilled criteria for admittance to geriatric care. A decision support tool had on beforehand been adapted for each condition respectively [17]. Following the outcome of the decision support tools, included patients could be transported to either of the alternative pathways: acute care centre or geriatric ward located at the same community hospital; or the standard pathway to the ED at an acute hospital [11].

### Rationale and aim

In short, the decision system in the previous RCT study was successful in terms of feasibility and patient satisfaction [11, 18], however, the included patients’ subsequent healthcare resource use and patient pathways have not been studied. This is essential, as changing initial destination could potentially affect subsequent inter-hospital transfers. A minimisation of inter-hospital transfers could be beneficial for the patients, as each transition comprises a risk for e.g. miscommunication between settings or lack of information to the patient [19]. It is also likely that fewer inter-hospital transfers decrease resource use, such as healthcare utilisation and costs [20]. To improve the understanding of healthcare patterns and resource use, and to facilitate healthcare planning for older patients, the aim of this study was to map and describe patient pathways, healthcare utilisation and costs following ambulance transportation to alternative healthcare providers.

## Methods

### Study design

The present study is based on the previous RCT in Stockholm, Sweden, in which older patients were transported to alternative levels of healthcare, based on a decision system. The RCT has been described in detail elsewhere [11, 17]. In this study, the design was descriptive and observational, based on register data. Patients that had been assessed for level of care were followed over a 10-day period. The length of follow up was set to 10 days as mean number of days at the geriatric ward was 9.5 [11]. Ethics approval was granted by the Regional Ethical Review Board in Stockholm (reference number 2016/2373–31/5). Informed consent for this register-based follow-up study was waived by the ethics committee.

### Study setting

Two ambulance companies in Stockholm Region were included in the RCT, one constituted the intervention group and the other the control group. The intervention group received training and access to decision support tools to guide the decision, together with medical assessment, whether to convey the patient to one of three providers; an acute care centre, a geriatric ward, or an ED. The control group followed standard protocol [11].

In Stockholm there are seven EDs located at acute hospitals. In the context of Swedish health services, geriatric units consist of multiprofessional teams, targeting older adults with deteriorated health conditions, or for rehabilitation or continued treatment after acute inpatient care [21]. In Stockholm, geriatric specialist care is provided at 12 geriatric units. Geriatric units can be located either independently or within other departments, both at community- and acute hospitals. The number of geriatric beds have increased over the last years, and in 2017 there were 1015 geriatric beds in the Stockholm Region [22]. The geriatric ward in this setting was located at a community hospital, in which the participating acute care centre, and a laboratory and radiology department were also located [11]. The acute care centres in Stockholm are primary care outpatient clinics and treats patients with acute, but less severe health conditions [23].

### The decision system

After identification of any of the 11 included conditions, patients in the intervention group were assessed with the decision support tool. In case of urgent symptoms, the tool guided the decision to conveyance to ED. The next step was assessment of whether vital parameters were within reference, any deviance from references meant conveyance to the ED. Otherwise, severity level was assessed and depending on severity level, the tool indicated conveyance to either ED, geriatric ward or acute care centre. Every decision in the ambulance to convey patients directly to geriatric care or acute care centre was taken in concordance with the physician in charge at the geriatric ward through initial telephone contact by the EMS [11].

### Study population

**Fig. 1:**
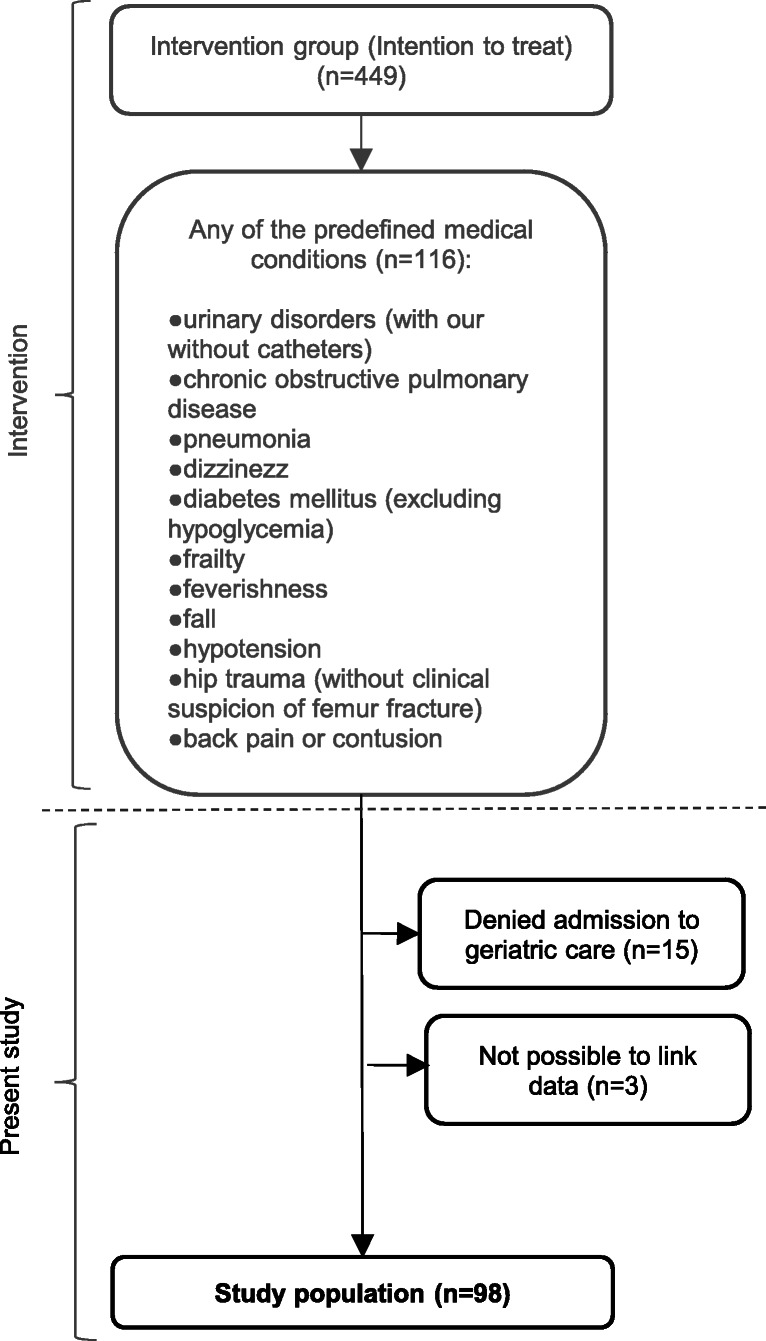
Scheme of inclusion of study population

This study describes the patients that were assessed with the decision support tool and conveyed accordingly. This implies that all patients had first been included in the RCT were patients ≥65 years old, living in a specific catchment area, called for an ambulance between 8 am and 10 pm, and prioritised as priority 2 or 3 (urgent, but not life-threatening conditions). Only patients that had any of the predefined conditions and consequently assessed with the decision system were eligible for inclusion in this study (*n* = 116) (Fig. 1). All patients included in this step had been conveyed to any of the three types of destination. Exclusion criteria for this study were denied admission to the geriatric ward (*n* = 15), due to lack of hospital beds or closed radiology department (during summer months), and restrictions in linking data to the register (*n* = 3). The remaining 98 cases constitute the study population. Some individuals appeared multiple times in the intervention (*n* = 18), therefore, in this study, data were handled as cases and followed based on initial event.

### Data

Information on cases were derived from several sources. Data from the intervention were linked to the administrative Stockholm regional healthcare database, which entails individual level information on public and private healthcare financed by the region, and is pseudonymised. Information on costs stems from the national cost per patient database, provided by the Swedish Association of Local Authorities and Regions [23], and regional price lists from Stockholm Region [24]. Data was managed with SAS version 9.4 [25] and the networkd3 package in R software [26, 27].

The index event for the period was ambulance transportation. References to inpatient care includes all hospitalisations, regardless of hospital type. For hospital stays that exceeded 10 days, the stay was cut-off. Outpatient care included visits at specialised care, advanced home care, and primary care, including home-based healthcare. Outpatient care was limited to physical visits by physicians, nurses (including different specialisations) or assistant nurses.

### Outcomes and analyses

Case-mix of the study population are presented as age, sex, Charlson index and history of healthcare utilisation. Charlson index is an index to predict death from comorbid disease, where a higher score indicates a higher risk [28]. History of healthcare utilisation includes all inpatient and outpatient care over 6 months prior to index event.

Descriptive analyses of patient pathways are presented in a Sankey diagram, starting at index event to receiving provider. In the pathway analysis, each transition is an episode, with no consideration taken to length-of-stay at each provider. All subsequent inpatient episodes, regardless of provider, were aggregated into continuous spells. The analysis demonstrates the paths of transitions within and between hospitals for the first continuous inpatient spell. Cases that were discharged, admitted over 10 days, or died, end their spell with last episode respectively. Percentages for each transition represents percent of total sample.

Resource utilisation is described using number of admissions to hospital, hospital days, outpatient visits and direct healthcare costs in total and by receiving provider, for the 10-day period. As we lacked information of time of the day the events occur, all events on index day were included. Mean, median, standard deviation, interquartile range, and minimum and maximum values are presented as measures of distribution. Cases that died during the period were excluded from the estimation.

Cost calculations of direct costs, from the perspective of the healthcare system, included costs for ambulance transportation, outpatient visits and inpatient care. For inpatient- and specialised outpatient care, aggregated means by hospital type and medical field for patients ≥65 years old in Stockholm region were applied. For all other outpatient care and ambulance transportation, fixed unit costs were applied for cost calculations. All prices were converted with the regional index into 2010 years prices and converted to euros with the 2010 average exchange rate (1 € = 9.54 SEK**)**.

## Results

The study population had a large age span with a mean age of 83 years, but a total range from 65 to 104 years (Table 1). The majority had comorbidities. History of healthcare utilisation indicated a large variation in use of healthcare resources, with a median of 2.5 outpatient visits per month. Almost all cases had minimum one outpatient visit, and one third were admitted to hospital, over the 6 months period prior to index event.

**Table 1:** Case-mix of the study population (*n* = 98)

	% (n)	Mean (±std^**a**^)	Median (min-max)
**Age**		83 (±8)	83 (65–104)
**Sex**			
Women	59 (58)		
Men	41 (40)		
**Charlson index**		1.5 (±1.6)	
Index groups			
0	31 (30)		
1–2	54 (53)		
3+	15 (15)		
**History of healthcare utilisation (6 months)**			**Median (IQR**^**b**^**)**
Admissions to hospital	36 (35)	1 (±1)	0 (0–1)
Hospital days		6 (±13)	0 (0–6)
Outpatient visits	97 (95)	34 (±54)	15 (6–31)

^a^*std* = standard deviation

^b^*IQR* = Interquartile range: 25th to 75th percentile

### Healthcare pathways

**Fig. 2:**
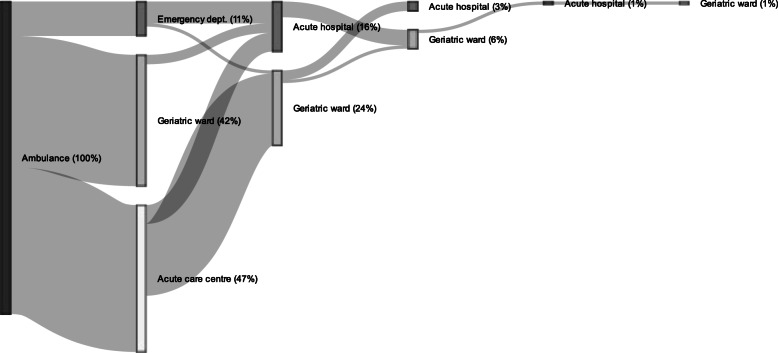
Routes of transition for the first continuous inpatient spell. The figure illustrates patient pathways over 10 days. All inter- and intra-hospital transfers in the first continuous inpatient spell, starting with ambulance transportation to each receiving provider respectively, are included. Percentages represent percent of total sample

The routes of transition for the first continuous inpatient spell are presented in Fig. 2. The maximum number of transitions within the 10-day follow-up period was 5 and the minimum was 1. Three percent died during the follow-up period. At the end of the period, about one third of the cases were still in hospital.

Almost 90% were transported to the acute care centre or geriatric ward. More than half of the cases that arrived to the acute care centre were later admitted to hospital, mainly to the geriatric ward, but also a smaller proportion to the acute hospital. However, 17% were discharged after initial assessment (number not shown in figure).

The vast majority that arrived to the geriatric ward either stayed there over the whole study period or until discharged. Additionally 24% of the cases were admitted to the geriatric ward after initial assessment at another destination. Only a minor part were transferred from the geriatric ward to the acute hospital in their first transition after arrival.

The majority of the cases at the ED were admitted to the acute hospital, a minor proportion to the geriatric ward, and the rest were discharged.

### Resource utilisation

Inpatient care comprised 92% of total cost, and inpatient care at the geriatric ward dominated as largest cost driver with 60% of total cost.

The overall median case had a hospital stay of 6 days, 2 outpatient visits, and a cost of roughly 4000 euro over the 10-day follow-up period (Table 2). Arriving at the ED indicated a shorter hospital stay, but a higher cost, whereas the arrival destination geriatric ward showed a longer stay, but a lower cost than the ED. The lowest median, but widest range, of costs was held by cases arriving to the acute care centre. The large spread in number of outpatient visits and inpatient days demonstrates the diversity of patients’ needs.

**Table 2:** Resource utilisation over 10 days subsequent to index event for surviving cases

	Mean (std^**a**^)	Median (IQR^**b**^)	Min-Max
**In total (*n*** **= 95)**			
Admissions to hospital	1 (±1)	1 (1–1)	0–4
Hospital days	6 (±4)	6 (3–10)	0–10
Outpatient visits	3 (±3)	2 (1–3)	0–19
Cost (€)	4382 (±2732)	3962 (2502–5809)	343–12,674
**By receiving provider**			
**Emergency department (*n*** **= 11)**			
Admissions to hospital	1 (±1)	1 (0–1)	0–2
Hospital days	4 (±4)	4 (0–10)	0–10
Outpatient visits	4 (±6)	1 (1–5)	1–19
Cost (€)	5248 (±2676)	5137 (3345–8171)	515–8812
**Geriatric ward (*n*** **= 40)**			
Admissions to hospital	1 (±0)	1 (1–1)	1–3
Hospital days	7 (±3)	8 (5–10)	1–10
Outpatient visits	2 (±2)	1 (0–2)	0–9
Cost (€)	4366 (±1680)	4440 (3205–5087)	1709–9091
**Acute care centre (*n*** **= 44)**			
Admissions to hospital	1 (±1)	1 (0–1)	0–4
Hospital days	5 (±4)	5 (0–10)	0–10
Outpatient visits	3 (±2)	2 (1–4)	0–10
Cost (€)	4180 (±3443)	3726 (795–6230)	343–12,674

^a^std = standard deviation

^b^IQR = Interquartile range: 25th to 75th percentile

## Discussion

Only 11% of the cases in the present study were conveyed by ambulance to the ED, which implies that almost 90% avoided ED attendance. Our results show that older patients transported directly, based on the decision system, to a geriatric ward at a community hospital to a vast majority stayed there until discharged. This supports that the geriatric ward as receiving provider could potentially minimise inter-hospital transitions, and indicates appropriate decisions in the ambulance. This is an important aspect, as older patients are vulnerable in transitions within or between settings, as each transfer comprises a risk for adverse effects [19].

In contrast, over 50% of the cases arriving at the acute care centre were admitted to hospital. The large proportion of hospitalisations among these cases could indicate a weakness of the decision system, as acute care centres are primary care providers that treat patients with minor acute conditions not in need of inpatient care [23]. However, in this study, it is difficult to draw conclusions, because in this particular setting there was a pre-existing collaboration between the units at the community hospital, which allowed patients to first be assessed at the acute care centre and thereafter directly admitted to the geriatric ward. Therefore, more detailed analysis regarding appropriate receiving provider for these cases is recommended. Nevertheless, 17% of the cases conveyed to the acute care centre were discharged after initial assessment, which is approximately the same proportion as has previously been identified as potential candidates for primary healthcare in the ambulance [29].

The design of our study does not allow for cost and/or outcome comparisons of alternative pathways. Nevertheless, there are examples of initiatives of prehospital conveyance with some improved outcomes, such as lower mortality rate [12], fewer secondary transfers [13], shorter time to admission to hospital ward [10], fewer subsequent emergency calls and ED attendance [9], and patient satisfaction [9, 18]. This reveals that there are potential benefits with prehospital conveyance and decision systems to assess appropriate healthcare provider. Evidence on cost consequences of prehospital conveyance to community hospitals are scarce. However, alternatives to acute hospitals, such as care homes, special units, hospital-at-home services or community hospitals can, for certain older patients, be safe and possibly lower healthcare costs [30]. At the same time, neither cost effectiveness nor cost efficiency has been observed in studies comparing community hospitals to acute hospitals in care of older patients [31, 32]. As there is a potential to improve outcomes and lower healthcare costs with alternative pathways, we recommend studies with comparable groups for older patients assessed with the decisions system in order to draw conclusions regarding costs and outcomes.

### Strengths and limitations

This study is unique in the sense that both healthcare pathways and economic consequences of the decision system are being mapped. It is a strength to use register data, as it enables following the patient through their chain of care and throughout different levels of care in the healthcare system. Inter-facility transfers are difficult to address without linking data to registers; nevertheless, they are important in order to address the quality of a decision system [33]. Presentation of healthcare pathways and resource use gives context to the patient group and illustrates the complexity of healthcare need. Consequently, this reveals where potential improvements could be targeted and facilitates healthcare planning and decision-making.

However, there are some drawbacks of the study. Firstly, multiple inclusion of some individuals results in non-independence of observations, therefore results should be interpreted with caution. Furthermore, as we lacked information on time of the day in the registers, we could not distinguishing order of events for same date, which lead to inclusion of all events on index day and presumably an overestimation of resource use. Neither did we have information on living situation or home care provided by the municipality, which presumably effects resource use, as availability of home care could have an effect time of discharge. To minimise this effect follow-up time was limited to 10 days, as mean number of days at the geriatric ward was previously reported as 9.5 days [11]. Another limitation is that we have not addressed possible complications which could have an effect on length of stay. Patient suffering from complications consequently need more healthcare. Information regarding complications, as well as causes of death for deceased individuals would have provided valuable information when addressing the appropriateness of the decision system. However, cause of death was not addressed due to the low number of deceased individuals and complications was not addressed due to limited access of data. Additionally, cases that were excluded have not been studied in detail, therefore, the risk of selection bias cannot be ignored.

The results of the study may not be generalizable to a population distinct from the urban context with a similar setting. Even in Sweden, the structure and organisation of geriatric care has a history to differ across the country [34]. Additionally, availability of hospital beds at geriatric clinics are finite and access linked to admissions should be studied in more detail and in close collaboration with the units. In some places, geriatric care is to a large extent provided as home care, an aspect that would both have an effect on ambulance conveyance to geriatric care, as well as discharge rates. Unfortunately, this could not be addressed in this study due to limited access of data. Nevertheless, we believe that our results could contribute in developing a structure for acute geriatric care and improve elders’ pathways within the healthcare system, regardless of setting.

## Conclusion

With the growing healthcare need, alternative care pathways for elderly have potential to minimise pressure at emergency departments and number of transitions between providers. At best, it can even lower healthcare costs with quality unchanged or improved. The findings support the appropriateness of a decision system for direct admittance to community hospital geriatric care for older adults with certain conditions, which signals that not all patients are in need of the services at the emergency department. However, as patients conveyed to acute care centre primary care to a large extent were admitted to hospitals, the need of inpatient care for these patients cannot be ignored and ought to be addressed in more detail with regards to appropriate level of care.

## Data Availability

The data that support the findings of this study are available from the Healthcare Committee Department at Region Stockholm, the Ambulance Medical Service in Stockholm and the Swedish Association of Local Authorities and Regions but restrictions apply to the availability of these data, which were used under license for the current study, and so are not publicly available. Data are however available from the authors upon reasonable request and with permission from the Ethical Review Board and the organisations responsible for data respectively.

## Abbreviations

ED: Emergency department
EMS: Emergency medical services
RCT: Randomised controlled trial
